# BioProspecting: novel marker discovery obtained by mining the bibleome

**DOI:** 10.1186/1471-2105-10-S2-S9

**Published:** 2009-02-05

**Authors:** Peter L Elkin, Mark S Tuttle, Brett E Trusko, Steven H Brown

**Affiliations:** 1 Center for Biomedical Informatics, Mount Sinai School of Medicine, New York, NY, USA; 2 Department of Medicine, Vanderbilt University, Nashville, TN, USA

## Abstract

BioProspecting is a novel approach that enabled our team to mine data related to genetic markers from the New England Journal of Medicine (NEJM) utilizing SNOMED CT and the Human Gene Onotology (HUGO). The Biomedical Informatics Research Collaborative was able to link genes and disorders using the Multi-threaded Clinical Vocabulary Server (MCVS) and natural language processing engine, whose output creates an ontology-network using the semantic encodings of the literature that is organized by these two terminologies. We identified relationships between (genes or proteins) and (diseases or drugs) as linked by metabolic functions and identified potentially novel functional relationships between, for example, genes and diseases (e.g. Article #1 ([Gene - IL27] = > {Enzyme - Dipeptidyl Carboxypeptidase 1}) and Article #2 ({Enzyme - Dipeptidyl Carboxypeptidase 1} < = [Disorder - Type II DM]) showing a metabolic link between IL27 and Type II DM). In this manuscript we describe our method for developing the database and its content as well as its potential to assist in the discovery of novel markers and drugs.

## Background

### "The best way to predict the future, is to create it" – Peter Drucker, Harvard University

The Bioinformatics and Genomics communities have had great success in the past several years in the discovery of the genetic basis of Mendelian disorders [1]. This success has been achieved mainly via the use of computing tools delivered by the field of Bioinformatics [2]. The bioinformatics research community employs technology, and pathological and genetic standards to assist in the linkage of genes and disorders [2]. Unfortunately, many diseases are not easily traced to a single genetic variation and the true origin of a disease is a complex interplay of genetic variations, environmental factors, "lifestyle" characteristics, with some stochastic processes [3].

To advance medical science and healthcare, a broader understanding of genetic markers and their relationships to each other is needed [4]. Our project was an attempt to "discover" markers and later research, linkages between variants discerned through the mining of the medical literature.

*"It is the responsibility of those of us involved in today's biomedical research enterprise to translate the remarkable scientific innovations we are witnessing into health gains for the nation. ... What novel approaches can be developed that have the potential to be truly transforming for human health? *[5]* Elias Zerhouni, M.D., 2005*

#### NEJM

The New England Journal of Medicine is widely considered one of the most influencial peer-reviewed medical journals in the world [6]. It is the oldest continuously published medical journal. The journal was inaugurated in 1812 as the New England Journal of Medicine and Surgery [7]. In 1928 the NEJM took on its present name after one hundred years as The Boston Medical and Surgical Journal.

The website for the George Polk Award noted in its 1977 award that the *New England Journal of Medicine *"provided the first significant mainstream visibility for a publication that would achieve enormous attention and prestige in the ensuing decades." [8]

The journal publishes widely cited editorials, papers on original research, review articles, correspondences and case reports. The journal consistently has the highest impact factor of the journals of clinical medicine (including the *Journal of the American Medical Association*, and *The Lancet*); in 2006, the impact factor was 51 and according to "Journal Citation Reports" was the first research journal to surpass 50.

The *NEJM *provides on-line (electronic) access dating back to 1994. Our study utilized all electronic data for the 27,000 on-line articles published from January 1994 through December 2006.

#### SNOMED CT (**S**ystematized **No**menclature of **Med**icine)

SNOMED CT (SCT) is a large-scale ontology used for the description of current medical and health community knowledge [9]. We utilized SCT due to its value for the functional purposes of computation from the medical literature and the issues related to maintaining and delivering it for those purposes. SCT is maintained by the College of American Pathologists (CAP) and is designed to represent all health and medical domains. Although very broad with over 360,000 concepts and 1.2 million relations it does not provide complete coverage of all medical content. The SCT modeling has been performed over more than a forty-year period and in the late 1990s SNOMED RT was merged with another large ontology, the Reed codes v3, developed in the United Kingdom.

Ontologies, to be useful for computation for practical tasks, need to be precise in two ways; firstly, they need to match as closely as possible our understanding of the natural world we wish to deal in. Ontologies need to be accurate and closely follow human understanding otherwise they create confusion in their design and their use. This means the ontology needs to be constructed with very close attention to the meanings of the terms used in it. Hence the names used in the ontology need to represent closely the understanding we have of the real world [10].

Secondly the variety of linguistic "usage" of those terms needs to be explored to uncover the diversity of semantic roles of the terms and ensure that only those roles that are useful are included in the ontological modeling, and to remove ambiguity in the use of those roles. Precision of definition is particularly important in establishing the relationships between elements and identifying the fundamental atomic elements and how they combine or "fit together" systematically to form compositional expressions. Ontologies also consist of abstractions with each level of the ontology being more abstract as one moves up the hierarchy. This structure allows us to talk about the world at the different levels of abstraction we would experience outside the realm of the "machine". In using ontology for computation there are two basic forms of abstraction available, aggregation and generalization. Generalization hierarchies are used throughout SCT as the basic mechanisms for relating content. Aggregation hierarchies on the other hand have not been used properly but rather transposed so that they appear like generalization hierarchies.

SCT provides coverage for diagnoses, findings, procedures and testing. In one study SCT provided 92.3% coverage of common medical problems seen at the Mayo Clinic [11]. It has been used in subsequent studies for electronic quality monitoring.[12]

#### HUGO

The **Human Genome Organisation **(HUGO) is an organization involved in the Human Genome Project, a project about mapping the human genome. HUGO was established in 1989 as an international organization, primarily to foster collaboration between genome scientists around the world. The HUGO Gene Nomenclature Committee (HGNC) is one of HUGO's committees and it aims to assign a unique gene name and symbol to each human gene.

## Results

### Novel Relationships

There are 574 metabolic functions that were used to link to Genes, Proteins, Disorders, and Drugs. See Tables 1, 2, 3, 4, 5

**Table 1:** Links between genes and disorders through a metabolic function

**DISTINCT (Gene-Disorder Pairs)**	**DISTINCT (Gene)**	**DISTINCT (Disorder)**
GENE [HUGO HGNC Database] – DISORDER (14,293,089)	GENE [HUGO] (2,244)	DISORDER (8,076)
GENE [20889005] – DISORER (16,135)	GENE [20889005] (3)	DISORDER (7,728)
GENE [67271001] – DISORDER (151,329)	GENE [67271001] (24)	DISORDER (8,064)
GENE [8116006] – DISORDER (72,082)	GENE [8116006] (13)	DISORDER (8,059)

Total	-	14,532,635

**Table 2:** Links between drugs and disorders through a metabolic function

**DISTINCT (Drug-Disorder)**	**DISTINCT (TERM1)**	**DISTINCT (TERM2)**
DRUG-DISORDER (11,312,477)	DRUGS (1,783)	DISORDER (8,076)

		

**Protein-Disorder**	**Protein**	**Disorder**

PROTEIN [41175001] – DISORDER (5,576,445)	PROTEIN [41175001] (890)	DISORDER (8,076)
PROTEIN [61321005] – DISORDER (144,741)	PROTEIN [61321005] (24)	DISORDER (8,057)
PROTEIN [88878007] – DISORDER (5,694,011)	PROTEIN [88818007] (878)	DISORDER (8,076)

Total – 11,415,197		

**Table 3:** Links between proteins and drugs through a metabolic function

**Protein-Drug**	**Protein**	**Drug**
PROTEIN [41175001] – DRUG (1,270,905)	PROTEIN [41175001] (890)	DRUG (1,783)
PROTEIN [61321005] – DRUG (33,652)	PROTEIN [61321005] (24)	DRUG (1,780)
PROTEIN [88878007] – DRUG (1,315,141)	PROTEIN [88818007] (878)	DRUG (1,783)

Total – 14,034,895		

**Table 4:** Links between genes and drugs through a metabolic function

**DISTINCT (TERM1-TERM2**	**DISTINCT(TERM1)**	**DISTINCT (TERM2)**
GENE [HGNC]-DRUG (3,314,327)	GENE [HUGO] (2,244)	DRUG (1,783)
GENE [20889005]-DRUG (3,709)	GENE [20889005] (3)	DRUG (1,749)
GENE [67271001]-DRUG (35,274)	GENE [67271001] (24)	DRUG (1,782)
GENE [8116006]-DRUG (15,931)	GENE [8116006] (13)	DRUG (1,782)

Total – 3,369,241		

Grand Total – 54,664,445

Known relationships identified within NEJM articles

**Table 5:** Summary data including novel and non-novel links

**BASE ON DISTINCT (TERM1 – FUNCTION – TERM2)**	
DRUG_DISORDER	(115,602,787)
GENE_DISORDER	(51,833,042)
GENE_DRUG	(5,831,303)
PROTEIN_DISORDER	(213,884,964)
PROTEIN_DRUG	(26,554,764)
Total	**413,706,860**

We identified 468,371,305 relationships within the 27,000 NEJM full text articles available online from the Massachusetts Medical Society. The dates of the articles ranged from 1994 to 2006. Of these over 54 million (11.7%) were potentially novel relationships, having never been mentioned together in any one NEJM article and over 413 million were previously known. Of the over 66 million relationships identified between Genes and Diseases, over 14 million (21.9%) were potentially novel. This degree of novelty represents a powerful opportunity to capitalize on synergies between articles in the medical literature. Additionally, should we "encode" other electronic journals, with different editorial perspectives, and introduce them into the broader universe of medical literature the benefits are expected to be even greater.

An example of two articles that "synergized" to link the "Fyn" gene with Myocarditis using their common association with kinase activity were:

*Predicting Progression – ZAP-70 in CLL*

Terry J. Hamblin, M.D.

*"In T-cell signaling, collaboration between ZAP-70 and the ****other Src kinases***, *Lck and ****Fyn***, *leads to the activation of downstream signaling pathways, including nuclear factor ...κB and mitogen-associated protein kinase, leading to cellular proliferation."*

Which linked with:

*Novel Role for Integrin-Linked Kinase in Modulation of Coxsackievirus B3 Replication and Virus-Induced Cardiomyocyte Injury*

Mitra Esfandiarei, Agripina Suarez, Ansel Amaral, Xiaoning Si, Maziar Rahmani, Shoukat Dedhar, Bruce M. McManus

*'Recently, more consideration has been given to the role of signaling pathways in pathogenesis of enteroviral ****myocarditis***, *providing new platform for identifying a new potential therapeutic target for this*, *so far, incurable disease.".... "Here, we report on regulation of virus-induced Akt activation by the ****integrin-linked kinase****in infected mouse ****cardiomyocytes****and HeLa cells."*

**Figure 1:**
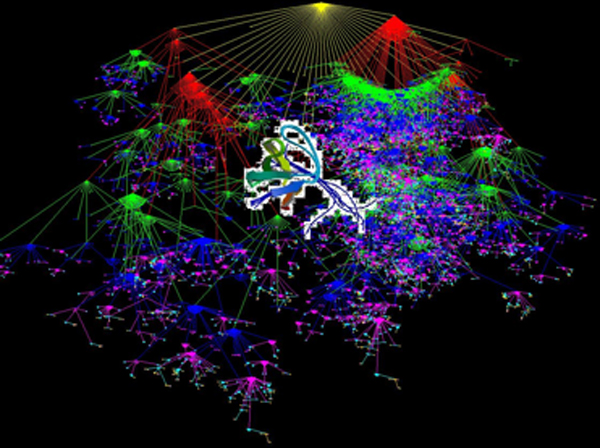
NMR study of the SH3 domain from FYN proto-oncogene tyrosine kinase complexed with the synthetic peptide P2L corresponding to residues 91–104 of the P85 subunit of the PI3-kinase, family of 25 structures. [13]

Figure 1 shows the connection between the Fyn gene and Myocarditis based on their connection to Tyrosine Kinase which is serving as a metabolic function associated with both the disease and the gene. The figure shows the disease hierarchy for Cardiovascular diseases from SNOMED CT.

**Figure 2:**
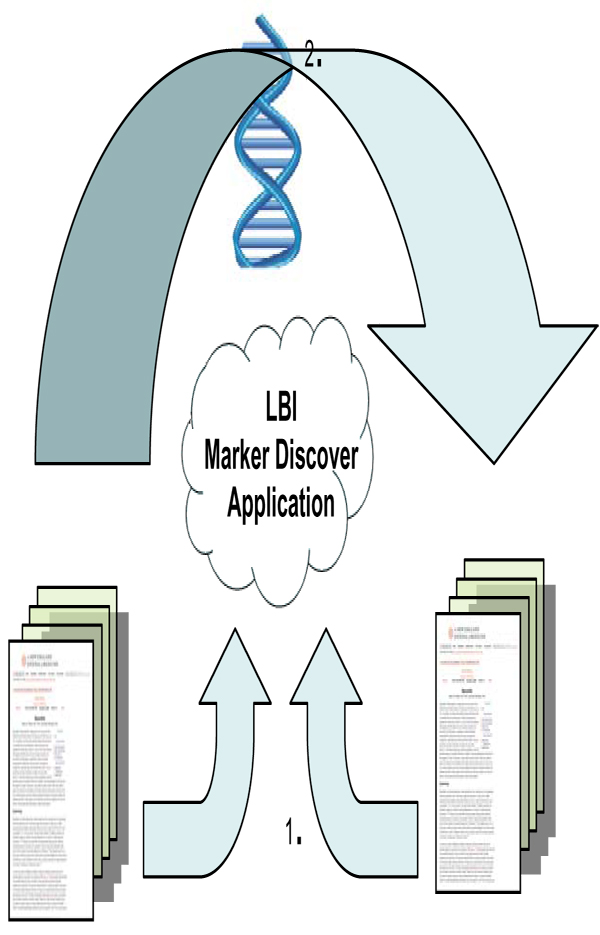
Illustrates the application parsing two journal articles, tying them to a common metabolic function and then "linking" the two articles together.

One could also envision the process as shown in figure 2:

## Conclusion

The medical literature is a vast repository of biomedical knowledge. Much of the richness of this resource is not in a form that is easily amenable to computational analysis. Add to this the fact that no medical professional can read all of the medical literature and Natural language processing has the potential to unlock the knowledge within the medical literature. In this experiment we used the information from the NEJM, one of the premier medical journals, to determine if novel relationships could be identified between Genes, Proteins, Drugs and Disorders. These relationships are indeed prevalent and hold the potential to increase our understanding of human disease.

The validation by example is illustrated in the identification of a gene "FYN" identified in tissue as a marker for Giant Cell Myocarditis, which was identified also in our database and would have been available from previous literature a full two-years in advance of its identification in tissue and publication. The database not only identified the relationship of the "FYN" gene to Giant Cell Myocarditis but also to Viral Myocarditis which would have otherwise been considered "not known". Identification of new relationships that have been validated in tissue is just one form of validation of the content of the database. We are currently looking at all of the genes related to more than three cancers. In this analysis we have identified 10 genes related to thirty or more cancers, 72 genes related to twenty or more cancers and 191 genes related to ten or more cancers. Perhaps the common basis for transformation of cells to malignancies has already been published but remains "hidden" within the vast amounts of biomedical literature.

Biomedical Informatics has the potential to help us to discover novel genetic linkage to human disease. This can lead to benefits in patient care with more rapid knowledge discovery and translational research empowering clinical implementation of personalized/individualized medicine.

## Methods

We parsed the full-text content of the New England Journal of Medicine (1994–2006) using our Multi-threaded Clinical Vocabulary Server (MCVS). The output of this technology creates an ontology-network using the semantic encodings of the literature that is organized by section of the article. In this effort we utilized a single Dell 2650 server with 8 GB of main memory and two processors. The configuration took one year to accomplish, but could have been done in 1/2 the time with two server and a quarter of the time with 4. The software is configured so that this scheme could continue indefinitely.

The indexing was done utilizing SNOMED-CT and the HUGO Ontologies. This provides robust indexing as SNOMED-CT has >370,000 concepts and >1,000,000 terms (in our lab we add another 790,000 terms to improve its clinical relevance) and HUGO has >26,000 human gene names. This concept based indexing represents a broad and consistent data infrastructure across articles from the literature.

We identified relationships between genes and functions, proteins and functions, diseases and functions and drugs and functions. Next, we matched these data sets across function, identifying functional relationships between proteins and diseases, for example. Further functional relationships were developed between genes and diseases, proteins and drugs, genes and drugs, and drugs and diseases. Next, we identified the disjoint sets where, for example, a gene and a disease match across function but have not been mentioned together in any previous NEJM journal article. Our goal was to identify synergy between articles within the literature indicating potential relationships between genes or proteins and drugs or diseases that have heretofore not been previously recognized. The database is searchable in all aspects such that a disease-oriented researcher could search the disease they are interested in and find all of the genes, proteins and drugs associated with that disease organized by function. Researchers could either access this information regarding known synergies (where the gene and disease have been mentioned in the same article) that verifies the utility of the algorithm or discontinuities (where the gene and disease have a functional relationship, however the two entities have never previously been mentioned in the same Journal Article). This may indicate the possibility of a novel relationship that can then be taken back to the bench for further definition, identification and research. Proteins and drugs, for example, that have a functional relationship but that have never been recognized to affect one another, may be substrate for further basic science analysis thereby leading to more rapid marker and treatment discovery. We see this as a potential method for improving the research productivity.

**Figure 3:**
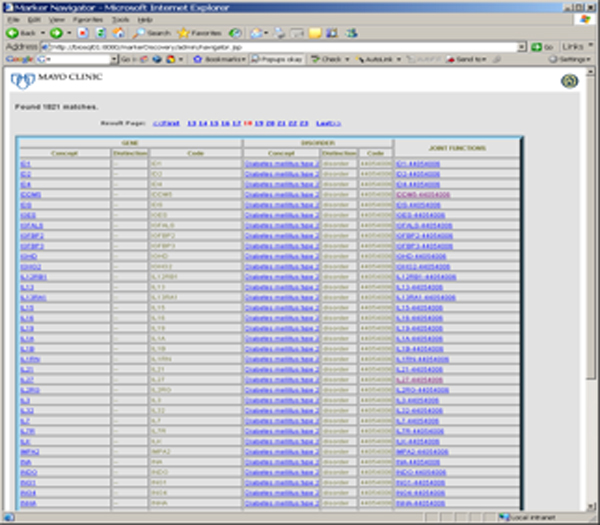
This screen depicts genes with a functional relationship to Type 2 Diabetes Mellitus. Clicking on the Gene searches pubmed with the gene name. Clicking on the disease name searches the Online Mendelian Inheritance of Man (OMIM) for that disease. Clicking on the function brings up the list of functions that link the Gene indicated with that Disorder of Interest (See Figure 4).

**Figure 4:**
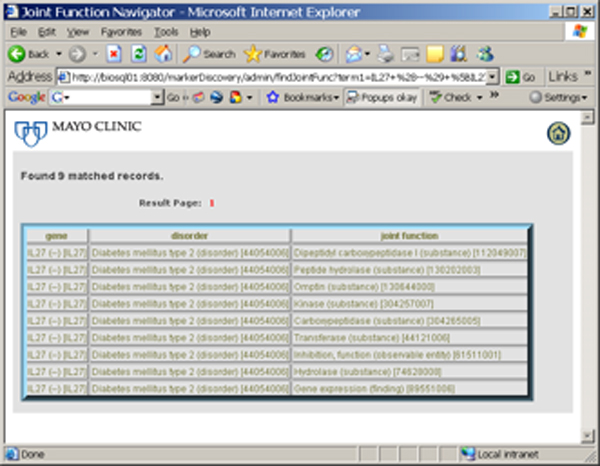
This screen shows the gene IL27 linked to the Disorder Diabetes Mellitus by metabolic functions such as Dipeptidyl carboxypeptidase I.

This program/exercise aims to support the development of novel clinical and translational methods that can encompass a wide range of techniques including new methods of phenotyping where one could use SNOMED CT to phenotype the patients described in this paper. New biomarkers for research are a potential output of this project (See Figure 1). This project may also benefit clinical informatics for longitudinal studies that aim to rapidly look at specific associations that may lead to either additional retrospective analysis or prospective clinical trials. The special deliverables of this project are also an interface where one can search the already recognized (concordant) relationships, and also disjoint or previously undocumented relationships between genes or proteins and drugs or diseases (See Figure 1). Free text searching will be available based on a gene, protein, disease, drug or function (See Figure 2). As organizing concepts are included, searching for classes (e.g. concepts like beta blockers for drugs or cardiovascular diseases) will be possible without having to articulate the individual sub-classes of information. See figures 3 and 4.

Should this "bear fruit" using the New England Journal of Medicine, other journals such as Science, Nature and Cell could be added to the corpora. The current collection holds the New England Journal of Medicine full text collection of over 27,000 articles. We did not survey the Science, Nature and Cell collections for an estimate of the addition to the collection, but we expect significant quality increases with the addition of these collections.

## Competing interests

The authors declare that they have no competing interests.

## Authors' contributions

PLE, MST, BET and SHB all contributed to the design study as well as the writing and review of the manuscript. PLE ran the study.
